# Resting state functional brain connectivity in child and adolescent psychiatry: where are we now?

**DOI:** 10.1038/s41386-024-01888-1

**Published:** 2024-05-22

**Authors:** Lucina Q. Uddin, F. Xavier Castellanos, Vinod Menon

**Affiliations:** 1 Department of Psychiatry and Biobehavioral Science, University of California Los Angeles David Geffen School of Medicine, Los Angeles, CA USA; 2 Department of Psychology, University of California Los Angeles, Los Angeles, CA USA; 3 Department of Child and Adolescent Psychiatry, New York University Grossman School of Medicine, New York, NY USA; 4 Nathan Kline Institute for Psychiatric Research, Orangeburg, NY USA; 5 Department of Psychiatry and Behavioral Sciences, Stanford University School of Medicine, Stanford, CA USA; 6 Department of Neurology & Neurological Sciences, Stanford University School of Medicine, Stanford, CA USA; 7 Wu Tsai Neurosciences Institute, Stanford University, Stanford, CA USA

**Keywords:** Diseases of the nervous system, Psychiatric disorders

## Abstract

Approaching the 30th anniversary of the discovery of resting state functional magnetic resonance imaging (rsfMRI) functional connectivity, we reflect on the impact of this neuroimaging breakthrough on the field of child and adolescent psychiatry. The study of intrinsic functional brain architecture that rsfMRI affords across a wide range of ages and abilities has yielded numerous key insights. For example, we now know that many neurodevelopmental conditions are associated with more widespread circuit alterations across multiple large-scale brain networks than previously suspected. The emergence of population neuroscience and effective data-sharing initiatives have made large rsfMRI datasets publicly available, providing sufficient power to begin to identify brain-based subtypes within heterogeneous clinical conditions. Nevertheless, several methodological and theoretical challenges must still be addressed to fulfill the promises of personalized child and adolescent psychiatry. In particular, incomplete understanding of the physiological mechanisms driving developmental changes in intrinsic functional connectivity remains an obstacle to further progress. Future directions include cross-species and multimodal neuroimaging investigations to illuminate such mechanisms. Data collection and harmonization efforts that span multiple countries and diverse cohorts are urgently needed. Finally, incorporating naturalistic fMRI paradigms such as movie watching should be a priority for future research efforts.

As around 75% of life-time psychiatric conditions emerge during childhood and adolescence, understanding the typical and atypical development of brain networks has become a primary focus of child and adolescent psychiatric neuroscience research. The study of neurodevelopmental psychopathology has been revolutionized by advances in network neuroscience enabled by the advent of resting-state functional magnetic resonance imaging (rsfMRI) [1]. From rsfMRI data, measures of functional connectivity (statistical interdependencies of blood oxygen level-dependent (BOLD) signals between brain regions) are computed that can reveal the intrinsic functional architecture of the brain [2]. During rsfMRI data acquisition, participants “rest”, without being required to engage in a specified cognitive task. As such, the procedure minimizes burden on participants, which has been a boon to researchers seeking to study the developing brain [3].

A number of principles have emerged from the several decades of rsfMRI research. First and foremost, we have learned that “resting state networks” or “intrinsic connectivity networks” are detectable in all brains, from healthy to psychiatric populations, from fetuses to advanced aging adults [4]. Information about age-related changes in hierarchical functional brain organization and reconfiguration of large-scale functional networks accompanying brain maturation has been obtained from analysis of rsfMRI data collected from participants across the human lifespan [5]. Another major insight is that the neural basis of many psychiatric conditions is less focal than previously believed, and that altered functional connectomes are observed across multiple (if not all) neurodevelopmental conditions [6]. This paradigm shift from focusing on individual brain regions as loci of dysfunction to analysis of large-scale brain networks and their interactions [7] was largely spurred by contributions from the rsfMRI literature.

In addition to traditional functional connectivity metrics, novel methods for examining functional connectivity dynamics are beginning to reveal more nuanced patterns of clinically-relevant brain network alterations [8, 9]. For example, analysis of brain dynamics, or time-varying changes in functional connectivity, has yielded the insight that youth with autism spectrum disorder (ASD) appear to exhibit alterations in the number of transitions between brain states compared with typically developing youth—findings that are plausibly linked to cognitive and behavioral rigidities that are strongly associated with the condition [10]. An important future direction necessary for further progress in applications of functional connectivity dynamics will be the resolution of ongoing debates surrounding appropriate analysis of these signals [11] (Table 1).

**Table 1:** Unresolved issues for the future of resting state functional connectivity in child and adolescent psychiatry.

Unresolved issue	Future directions
Artifacts due to participant motion	Advances in real-time monitoring and incorporation of novel data-denoising strategies
Measurement of brain dynamics	Cross-species validation studies; quantification of uncertainty
Need for more naturalistic data acquisition beyond resting state	Incorporation of movie-watching paradigms
Inconsistent brain network nomenclature	Adoption of best practices and tools for standardized reporting
Lack of diversity in population neuroscience datasets	Oversampling of individuals from minoritized sociodemographic groups, international collaborations, and transnational data sharing

The National Institute of Mental Health Research Domain Criteria initiative, which recognizes that dimensions of behavior can cut across traditional diagnostic categories, is a framework that aligns well with rsfMRI research and has already been widely implemented. While assessments of case-control differences in brain function have long dominated the field of clinical neuroscience, they can mask nuanced variations within diagnostic categories. Resting-state fMRI-derived metrics can be used as features in unsupervised machine learning approaches to reveal data-driven subtypes within heterogeneous clinical populations (Fig. 1). As an example showing great promise for parsing heterogeneity in ASD, recent evidence suggests that at least 2–4 distinct neurosubtypes exist in this increasingly prevalent neurodevelopmental condition [12]. These findings were made possible by a grass-roots data-sharing initiative known as the Autism Brain Imaging Data Exchange that aggregated multiple rsfMRI datasets collected independently by researchers around the world [13]. Other federally funded data-sharing consortia such as the longitudinal Adolescent Brain Cognitive Development Study have released thousands of rsfMRI datasets that can be used by researchers interested in neural precursors to substance use initiation and onset of mental illness in youth [14]. Features derived from rsfMRI and task fMRI data can be used to predict individual differences in cognitive performance, personality, and mental health [15]. We have learned from this emerging network neuroscience literature that atypical functioning of the salience (with key nodes in anterior insula and anterior cingulate cortex) and default mode (with key nodes in medial prefrontal and posterior cingulate cortex) networks is a transdiagnostic feature of many of the developmental conditions that are the focus of child and adolescent psychiatry [16, 17].

**Fig. 1:**
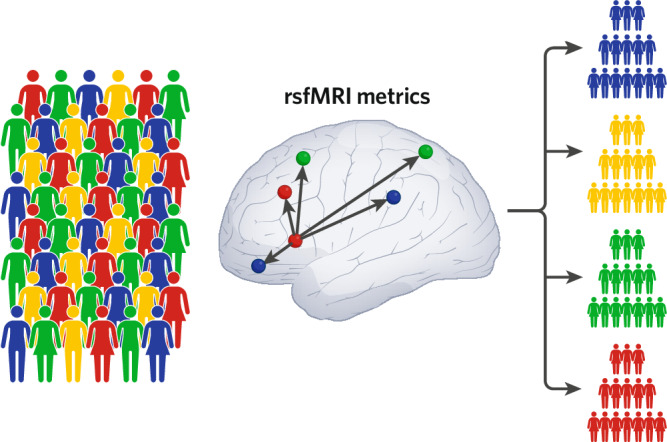
Unsupervised machine learning can be used to parse heterogeneity and identify subtypes within heterogeneous neurodevelopmental disorders based on rsfMRI functional connectivity metrics.

Resting-state fMRI has already begun to provide potential insights into the mechanisms and systems through which clinical treatments work. For example, a rsfMRI study in children with attention-deficit/hyperactivity disorder (ADHD) found that methylphenidate increased spontaneous neural activity in the nucleus accumbens, and the salience and default mode networks [18]. Moreover, methylphenidate-induced changes in spontaneous activity patterns in the default mode network were associated with improvements in intraindividual response variability during a sustained attention task. Additionally, methylphenidate-induced changes in brain state dynamics and functional connectivity between salience and default mode networks were also correlated with improvements in behavioral variability. Such investigations highlight the potential of rsfMRI to elucidate the neural mechanisms of treatment response in psychiatric disorders.

Of relevance to pathophysiology, aberrant intrinsic connectivity within large-scale brain networks can profoundly impact cognitive and affective processing systems. These disruptions may lead to significant difficulties with cognitive flexibility—the capacity to adapt cognitive processing strategies to face new and unexpected conditions—and emotional regulation, which is crucial for managing and responding to emotional stimuli adaptively. For instance, altered neural connectivity patterns can impair the brain’s ability to efficiently switch between cognitive tasks, thereby limiting cognitive flexibility. This phenomenon has been supported by empirical evidence showing that machine learning algorithms can distinguish between intrinsic and task-evoked functional brain network configurations with lower accuracy in children with ASD compared with neurotypical controls [19]. Furthermore, the discriminability of brain states has been correlated with the severity of restricted and repetitive behaviors in ASD, suggesting that a diminished modulation of brain states may underlie the behavioral inflexibility often observed in autism. Understanding relationships between dynamic network connectivity, discrimination of brain states, and clinical phenotype offers a promising avenue for advancing precision psychiatry [20]. This approach may eventually inform development of more effective, personalized interventions that target underlying network dysfunction to improve treatment outcomes for neurodevelopmental disorders.

The development of brain circuits revealed by resting state functional connectivity is influenced by a complex interplay of genetic, environmental, and physiological factors. Researchers have proposed several physiological mechanisms that may shape the development of resting state functional connectivity, but there is still incomplete understanding in this area. Resting-state functional connectivity reflects several factors including the amplitude of spontaneous low-frequency BOLD signal fluctuations and the strength of inter-regional coupling, which reflects both direct mono-synaptic and polysynaptic structural white matter pathways, as well as the history of co-activations among brain regions [21]. Physiological mechanisms proposed to shape resting state network development include experience-dependent Hebbian plasticity and neural reuse, as networks are shaped by shared activation history and learning; pruning and strengthening of synaptic connections during sensitive periods in development; developmental changes in neurotransmitters like Gamma-aminobutyric acid (GABA) and glutamate influencing excitation/inhibition (E/I) balance; and maturation of cognitive control networks facilitating transition from diffuse to focused activation patterns [22].

Several key principles further characterize typical and atypical development of functional connectivity [5]: (1) increased segregation of functional circuits from childhood to adulthood, with a shift from short-range to distinct long-range functional connectivity [23]; (2) reconfiguration of subcortical-cortical functional connections, including those linking basal ganglia and cortex, which are particularly important for learning and motivation [22]; (3) dynamic pruning, which helps rebalance and rewire functional connections [24]; (4) changes in E/I balance impacting local and global connectivity in typical and atypical development [25]; and (5) reconfiguration within and between large-scale networks reflecting modular changes with learning and experience [26].

These principles of functional brain development provide insights into how disrupted functional connectivity underlying psychiatric disorders may emerge. For example, incomplete pruning and segregation of functional circuits could result in diffuse overconnectivity which appears to be characteristic of childhood ASD [27]. Deficient maturation of cognitive control networks could contribute to executive dysfunction in ADHD. Aberrant reconfiguration of subcortical-cortical reward circuits could promote addictive disorders. Moreover, altered balance of E/I signaling has the potential to impact local processing and global connectivity in a transdiagnostic manner across developmental psychopathologies. Understanding trajectories of typical network development is key for elucidating points of divergence in atypical development underlying the onset of child and adolescent psychiatric disorders.

Overall, rsfMRI provides a powerful window into functional brain organization, development, and disrupted connectivity underlying psychiatric disorders. However, further research is critically needed to clarify the neurophysiological factors guiding typical and atypical network development. Recent elegant work examining relationships between rsfMRI and underlying neural activity in rodent brains using wide-field Ca^2+^ imaging suggests a strong degree of correspondence between large-scale functional brain network organization measured using these two methods [28]. Such convergence across dramatically distinct methods and spatial scales provides further support for the fundamental validity of rsfMRI.

Notwithstanding these promising developments, a number of challenges associated with rsfMRI data acquisition, analysis, dissemination, and interpretation must still be overcome to fulfill the promises of personalized child and adolescent psychiatry. At the data acquisition stage, of primary concern is the reality that younger individuals move much more in the scanner than older participants, creating artifacts in the acquired rsfMRI data that influence and distort functional connectivity estimates [29]. Consensus around how to best mitigate such motion-induced artifacts in rsfMRI data has yet to emerge, but efforts to monitor motion in real time have helped to improve data quality [30]. Importantly, many of the key principles surrounding age-related changes in functional connectivity that were initially reported as being confounded by motion artifacts have since been shown to remain significant after application of rigorous data processing pipelines controlling for the confounding influence of motion at both individual subjects and group levels [31]. Data acquisition protocols using multi-echo fMRI [32] and data processing pipelines incorporating strategies such as independent component analysis or wavelet despiking can accomplish some of these denoising goals [33].

Another set of challenges surround reliability and reproducibility of neuroimaging findings, including the inability to translate from group results to individually relevant, clinically-actionable outputs, and lack of mechanistic insights [34]. These issues are further compounded by non-standardized data processing approaches and inconsistent reporting conventions for network neuroscience results across all of cognitive and clinical neuroscience [35]. Many researchers are proponents of the “multiverse” strategy wherein multiple pipelines are evaluated on a given dataset simultaneously to arrive at consensus results. This strategy addresses the challenge of deciding which of many analytic pipelines to utilize [36].

Despite the availability of large rsfMRI datasets due to increasingly popular data-sharing efforts, a major obstacle to progress in precision psychiatry arises from the fact that systematic biases in current machine learning algorithms are overly tuned to majority populations and consequently fail to generalize to minority populations. For example, the performance of brain-behavior predictive models trained on rsfMRI datasets dominated by White participants breaks down when predicting phenotypes for Black participants [37]. These predictive models often reflect stereotypical profiles and fail to generalize to individuals with sociodemographic characteristics outside of the majority [38]. The reproducibility and generalizability of predictions of cognitive and mental health phenotypes for individuals belonging to minoritized groups is thus severely limited at this time. Going forward, these issues can and should be addressed using approaches such as stratified sampling developed in collaboration with epidemiologists and population scientists worldwide [39].

Most rsfMRI data acquisition paradigms require participants to lie still with eyes open or closed and “do nothing”. This can present a challenge to clinical and pediatric populations, particularly those with attention deficits. Movie-watching in the MRI scanner has emerged as an alternative “naturalistic paradigm” that can more readily be tolerated by young children, who exhibit reduced head motion when presented with this type of moderately engaging stimulus [40, 41]. Movie watching is also compatible with interesting analytic pipelines including intersubject correlations [42].

Additional future directions include cross-species and multimodal neuroimaging investigations to illuminate physiological mechanisms underlying developmental changes in resting state functional connectivity. Mechanistic studies of rsfMRI in non-human primates and rodent models are beginning to reveal conserved and unique patterns of brain network organization across species. rsfMRI connectivity mapping is now being combined with approaches including chemogenetic or optogenetic manipulations to uncover fundamental principles governing communication between brain regions [43]. This type of comparative neuroscience research is critical for providing avenues for perturbational studies that cannot be ethically conducted in humans.

Still, we know little about what developmental changes in rsfMRI functional connectivity reflect, as almost all of the cross-species investigations to date have been conducted in mature adult animals, who are either lightly anesthetized or extensively trained. Lengthy training is impractical in rodent animal models, given their exceedingly brief developmental periods. Thus, future basic science efforts to map developmental trajectories at the meso- and micro-scale will likely need to rely on innovative methods appropriate for developing non-human primates. Given the extraordinary investments of time and resources required, such initiatives will be best conducted through open-science collaborative venues, such as the Primate Data Exchange [44]. Ongoing work is leveraging the ability to study non-human primates along with human epilepsy patients with invasive electrophysiological methods in parallel with whole-brain MRI studies. Foundational work in adult animals and humans will be essential to tackling the even more challenging feat of doing so during development.

Overall, it is clear that several methodological and theoretical challenges must be addressed for rsfMRI approaches to continue to further the goals of personalized child and adolescent psychiatry. Based on the decades of rapid progress already achieved, we have reason to face these challenges with cautious optimism.
